# Overcoming immune-cell unresponsiveness in cancer

**DOI:** 10.1186/2051-1426-1-S1-P261

**Published:** 2013-11-07

**Authors:** Guenther Lametschwandtner, Monika Ermann, Ina Sternberger, Thomas Hesterkamp, Isabella Haslinger, Hannes Mühleisen, Manfred Schuster, Hans Loibner

**Affiliations:** 1 Apeiron Biologics, Vienna, Austria; 2 Evotec, Abingdon, UK; 3 Evotec, Hamburg, Germany

## 

Despite significant recent progress, cancer immunotherapy efficacies are still limited, since tumors can down-modulate and escape the immune system by various mechanisms. To overcome these limitations, we aimed to identify low molecular weight molecules that can enhance T cell reactivity in the context of otherwise insufficient or suppressive stimulation. In an HTS campaign, 80000 maximal diverse compounds were screened on anti-CD3/CD28 stimulated human PBMCs using enhancement of IL2 production as readout. The confirmed hit list contained 3 chemically independent series, which enhanced the production of inflammatory cytokines, especially IL-2, IFN-γ and TNF-α, from TCR-stimulated T cells, while lacking any agonist activity on unstimulated T cells. The compound-mediated enhancement of TCR-stimulation was also observed on the level of T cell activation markers like CD25, CD69, CD71 and CD40L. In line with these observations, compounds of these series enhanced the in vitro anti-tumor responsiveness of PBMCs against tumor cell lines, originating from melanoma (M21), neuroblastoma (LAN) and leukemia (K562). For MHC-positive tumor cells, refined analysis demonstrated that compounds enhanced CD8 T cell activation, resulting in selective tumor cell killing while compounds in the absence of immune cells did not affect tumor cell viability. In contrast, the PBMC-response against K562 cells, which is a commonly used NK cell target due to the lacking MHC-expression, was mainly mediated by NK cells. Moreover, compounds also enhanced IL-2 mediated NK cell proliferation and cytokine (IFN-γ, TNF-α) and effector molecule (Granzyme B, Perforine) secretion. The activities of these compounds were further confirmed in an antigen-specific ex vivo T cell stimulation assay of human PBMCs with Tetanus toxoid, which resulted in enhanced proliferation of T cells. In addition, enhanced IL-2 secretion by activated T cells led to consequently increased NK cell activation and proliferation in antigen-stimulated stimulated PBMCs. To enable future assessment of compound activities in murine tumor models in vivo, we have verified that compounds of all 3 series enhanced T cell activation and cytokine production of TCR-stimulated murine T cells, while they had no effect on unstimulated T cells. Together, we show here the identification of a novel class of low molecular weight compounds with drug-like properties that selectively enhance activation of T and NK cells in the context of antigen-specific and anti-tumor immunity with high potential for development of improved tumor immunotherapeutics.

